# Identification of a Heterozygous Mutation in the *TGFBI* Gene in a Hui-Chinese Family with Corneal Dystrophy

**DOI:** 10.1155/2019/2824179

**Published:** 2019-02-19

**Authors:** Qin Xiang, Lamei Yuan, Yanna Cao, Hongbo Xu, Yunfeiyang Li, Hao Deng

**Affiliations:** ^1^Postdoctoral Research Station of Basic Medicine, The Third Xiangya Hospital, Central South University, Changsha, China; ^2^Center for Experimental Medicine, The Third Xiangya Hospital, Central South University, Changsha, China; ^3^Department of Ophthalmology, The Third Xiangya Hospital, Central South University, Changsha, China

## Abstract

**Background/Aims:**

Corneal dystrophies (CDs) belong to a group of hereditary heterogeneous corneal diseases which result in visual impairment due to the progressive accumulation of deposits in different corneal layers. So far, mutations in several genes have been responsible for various CDs. The purpose of this study is to identify gene mutations in a three-generation Hui-Chinese family associated with granular corneal dystrophy type I (GCD1).

**Methods:**

A three-generation Hui-Chinese pedigree with GCD1 was recruited for this study. Slit-lamp biomicroscopy, optical coherence tomography, and confocal microscopy were performed to determine the clinical features of available members. Whole exome sequencing was performed on two patients to screen for potential disease-causing variants in the family. Sanger sequencing was used to test the variant in the family members.

**Results:**

Clinical examinations demonstrated bilaterally abundant multiple grayish-white opacities in the basal epithelial and superficial stroma layers of corneas of the two patients. Whole exome sequencing revealed that a heterozygous missense mutation (c.1663C > T, p.Arg555Trp) in the transforming growth factor beta-induced gene (*TGFBI*) was shared by the two patients, and it cosegregated with this disease in the family confirmed by Sanger sequencing.

**Conclusions:**

The results suggested that the heterozygous *TGFBI* c.1663C > T (p.Arg555Trp) mutation was responsible for GCD1 in the Hui-Chinese family, which should be of great help in genetic counseling for this family.

## 1. Introduction

Corneal dystrophies (CDs) belong to a group of hereditary and noninflammatory corneal disorders that give rise to corneal transparency loss and visual impairment due to the progressive accumulation of extracellular amyloid and nonamyloid deposits in different corneal layers [1–3]. Symptoms typically initiate in the first or second decade of life and slowly progress throughout life [1, 4]. Based on clinical features, pathologic exams, and genetic data, CDs were subcategorized as epithelial and subepithelial dystrophies, epithelial-stromal *TGFBI* dystrophies, stromal dystrophies, and endothelial dystrophies [5, 6]. Though a few are inherited as autosomal recessive forms, the majorities are inherited as autosomal dominant forms with a high degree of penetrance [4, 7]. Mutations in the transforming growth factor beta-induced gene (*TGFBI*, OMIM 601692), the solute carrier family 4 member 11 gene (*SLC4A11*, OMIM 610206), the collagen type VIII alpha 2 chain gene (*COL8A2*, OMIM 120252), the keratin 3 gene (*KRT3*, OMIM 148043), the keratin 12 gene (*KRT12*, OMIM 601687), the tumor associated calcium signal transducer 2 gene (*TACSTD2*, OMIM 137290), and the carbohydrate sulfotransferase 6 gene (*CHST6*, OMIM 605294) have been reported as being responsible for various CDs, in which the *TGFBI*-CDs are the most common [1, 8–12]. CDs are highly heterogeneous disorders, both clinically and genetically [1]. A certain subtype could result from different genetic defects, and mutations in a definite gene could also cause different subtypes [5, 13].

The human *TGFBI* gene, expression of which is induced by transforming growth factor-beta (TGF-*β*), encodes a TGF-*β*-induced protein (TGFBIp) which is located in the extracellular matrix [14]. Although it is believed to be involved in many cell processes including cell proliferation, differentiation, migration, adhesion, angiogenesis, and apoptosis, its function has not yet been completely understood [15]. *TGFBI* gene mutations are primarily involved in autosomal dominant inherited CDs characterized by the progressive accumulation of extracellular insoluble protein deposits within corneal tissue, which can present as amyloid, nonamyloid (granular), or both [15, 16]. Depending upon deposition features and locations in the corneal layers, disease-causing mutations identified in the *TGFBI* gene have been involved in various phenotypes, including Thiel–Behnke corneal dystrophy (TBCD, OMIM 602082), Reis–Bucklers corneal dystrophy (RBCD, OMIM 608470), Groenouw type I granular cornea dystrophy (CDGG1, also known as GCD1, OMIM 121900), Avellino corneal dystrophy (ACD, OMIM 607541), lattice corneal dystrophy types I and IIIA (LCD1, OMIM 122200 and LCD3A, OMIM 608471), and epithelial basement membrane corneal dystrophy (EBMD, OMIM 121820) [2, 4, 17]. Currently, *TGFBI*-CDs present as dominantly inherited monogenic forms with a high penetrance of 60–90% [16]. To our knowledge, at least 63 *TGFBI* gene mutations have been described as being involved in different subtypes of CDs [18].

This study identified a *TGFBI* gene heterozygous c.1663C > T (p.Arg555Trp) mutation in a three-generation Hui-Chinese family with CD.

## 2. Materials and Methods

### 2.1. Subjects and Clinical Evaluations

**Figure 1:**
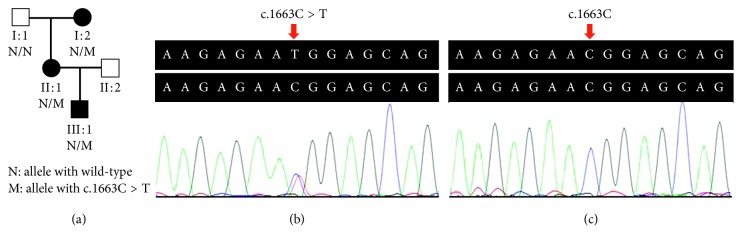
Pedigree of the Hui-Chinese family with GCD1 and sequencing analysis of *TGFBI* c.1663C > T mutation. (a) Pedigree of the GCD1 family. Squares and circles represent males and females, respectively. Solid symbols indicate patients, and open symbols indicate unaffected individuals. (b) The patient II : 1 with the heterozygous *TGFBI* c.1663C > T mutation. (c) The unaffected family member (I : 1) with the *TGFBI* c.1663C. GCD1, granular corneal dystrophy type I; *TGFBI*, transforming growth factor beta-induced gene.

Our study recruited a three-generation Hui-Chinese pedigree with CD (Figure 1(a)). Two experienced ophthalmologists performed ophthalmologic examinations on available members. The exams included best-corrected visual acuity (BCVA) assessed by the Snellen visual chart, slit-lamp biomicroscopy, optical coherence tomography (OCT), and confocal microscopy. All patients were diagnosed at the Third Xiangya Hospital of Central South University, Changsha, Hunan, China. This study followed the tenets of the Declaration of Helsinki and the guidance of the Institutional Review Board of the Third Xiangya Hospital of Central South University. After written informed consent was obtained, peripheral blood samples were collected from four available family members (I : 1, I : 2, II : 1, and III : 1). Genomic DNA (gDNA) was extracted from peripheral blood leukocytes using a phenol-chloroform extraction procedure [19, 20].

### 2.2. Whole Exome Sequencing and Data Analysis

Whole exome sequencing (WES) was performed by a commercial service from BGI-Shenzhen (Shenzhen, China) as previously described [21]. In brief, gDNA samples of two patients (I : 2 and II : 1, Figure 1(a)) were randomly fragmented into 150–250 bp by Covaris. DNA fragments were repaired by A-tailing reactions and were then ligated with adapters. Ligation-mediated polymerase chain reaction (PCR) was used to amplify size-selected DNA fragments. PCR products were further purified and enriched with an exome array. High-throughput sequencing was then performed for each captured exome library on the BGISEQ-500 platform according to the manufacturer's instructions. After variants called by BGISEQ-500 basecalling software, clean reads per sample were mapped onto the human reference genome (GRCh37/hg19) via the Burrows–Wheeler Aligner (BWA, v0.7.15). Picard tools (v2.5.0) and the Genome Analysis Toolkit (GATK) were used to mark and remove duplicate reads, respectively. All single nucleotide polymorphisms (SNPs) and insertions/deletions (indels) were called by the HaplotypeCaller of GATK (v3.3.0). All variants screened were further filtered using the Single Nucleotide Polymorphism database v141, the 1000 Genomes Project, and the National Heart, Lung, and Blood Institute Exome Sequencing Project 6500. Annotations for variants were performed by the SnpEff tool (http://snpeff.sourceforge.net/SnpEff_manual.html). Variants shared by two patients (I : 2 and II : 1) and occurring in known CD-causing genes were considered preliminary as candidate variants. Prediction for impacts caused by candidate variants was performed on Polymorphism Phenotyping v2 (PolyPhen-2), Sorting Intolerant from Tolerant (SIFT), and MutationTaster software. According to the American College of Medical Genetics and Genomics (ACMG) recommendations for interpretative categories of variants in Mendelian disorders, candidate variants were further classified as “pathogenic,” “likely pathogenic,” “uncertain significance,” “likely benign,” and “benign” [22].

### 2.3. Variant Validation

Potential CD-related pathogenic variants revealed by the WES were further confirmed in family members using Sanger sequencing and evaluated to determine whether it cosegregated with the disease phenotype in the family. Primers for PCR amplification were designed by Primer3 software (http://primer3.ut.ee/) based on human reference genome and synthesized by Sangon Biotech (Shanghai) Co., Ltd. (Shanghai, China) as follows: 5′-GACTGACGGAGACCCTCAAC-3′ (forward) and 5′-GATGTGCCAACTGTTTGCTG-3′ (reverse). PCR products were sequenced according to manufacturer's instructions on an ABI 3500 sequencer (Applied Biosystems, Foster City, CA, USA). The variant was compared with those of the 528 Chinese controls (including the 13 Hui-Chinese controls) of our in-house exome databases and the 1,943 Chinese controls without CDs from BGI in-house exome databases, as well as variant databases, the Exome Aggregation Consortium (ExAC) and the Genome Aggregation Database (gnomAD).

## 3. Results

### 3.1. Subjects and Clinical Assessment

**Figure 3:**
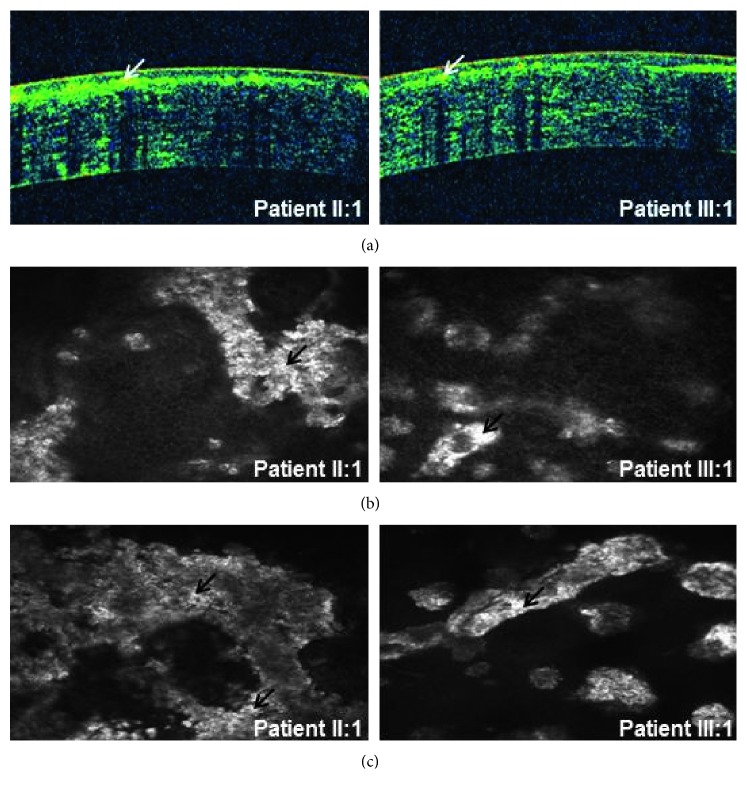
Optical coherence tomography and *in vivo* laser-scanning confocal microscopy images of patients in the Hui-Chinese family. (a) Optical coherence tomography scan of the patients (II : 1 and III : 1) demonstrated markedly increased reflectivity due to deposits within the superficial cornea. *In vivo* laser scanning confocal microscopy images showed many abnormal hyperreflective regions with irregular shapes existing in the basal epithelial cell layer (b) and the superficial stroma layer (c) of corneas in the patients (II : 1 and III : 1). Arrows indicate the abnormal and highly reflective deposits.

The transmission form of this family was consistent with autosomal dominant inheritance (Figure 1(a)). All patients in this family were diagnosed as GCD, and the clinical features of patients were presented below. The patient I : 2 was a 61-year-old woman who complained of bilateral poor vision and hyperdacryosis for about 20 years and presented with grayish-white granular opacities in her central corneas. Her preoperative vision was 20/667 OD and 20/1000 OS. A penetrating keratoplasty was performed in her left eye at the age of 61 due to impaired visual acuity. After penetrating keratoplasty, the visual acuity in her left eye was restored to 20/333 (anterior segment images not available). The patient II : 1 was a 41-year-old woman. Her vision was 20/33 OD and 20/40 OS. The patient III : 1 was a 19-year-old man. His vision was 20/33 OD and 20/50 OS. Slit-lamp examinations revealed bilaterally abundant multiple crumb-shaped and round grayish-white opacities in the central corneas of patients (II : 1 and III : 1, Figures 2(a) and 2(b)). OCT scan of patients (II : 1 and III : 1) demonstrated markedly increased reflectivity due to deposits within the corneas (Figure 3(a)). *In vivo* laser scanning confocal microscopy revealed many abnormal hyperreflective dots with sharp shapes primarily existed in the basal epithelial (Figure 3(b)) and superficial stroma layers (Figure 3(c)) in the corneas of patients (II : 1 and III : 1). Slit-lamp biomicroscopy showed unaffected corneas in the unaffected family member (I : 1, Figure 2(c)), while the family member II : 2 refused to have the examination with claimed unaffected vision.

### 3.2. Whole Exome Sequencing and Variant Validation

WES of the patient I : 2 and the patient II : 1 generated 26,402 Mb and 22,397.76 Mb raw data with an average sequencing depth of target regions 230.75× and 202.01×, respectively. A total of 108,186 and 107,154 SNPs, and 19,029 and 18,426 indels were separately obtained from patients I : 2 and II : 1. After filtering databases and functional analysis, only a heterozygous c.1663C > T (p.Arg555Trp) mutation in the *TGFBI* gene, shared by the two patients and previously reported for GCD1 [23], was proposed as the potential pathogenic mutation in this family. The mutation was predicted to be probably damaging, damaging, and disease-causing by PolyPhen-2, SIFT, and MutationTaster, respectively. By Sanger sequencing, the mutation was confirmed in all patients and absent in the unaffected family member (I : 1, Figures 1(b) and 1(c)), cosegregating with GCD1 in the family. It was also absent in the ExAC, gnomAD, and 2,471 Chinese controls from the in-house databases. According to the ACMG guidelines for variants interpretation, the heterozygous mutation was categorized as “pathogenic.” Taken together, the genetic and clinical data supported a diagnosis of GCD1 in this family.

## 4. Discussion

GCD1, also termed as classic granular CD or Groenouw CD, is one of the most common phenotypes of the *TGFBI* gene associated with CDs in China, which has an autosomal dominant trait [13, 24]. It is featured by the progressive accumulation of white or gray white granules in the corneal stroma, with onset usually in the first or second decade of life [7, 25]. In addition to superficial stroma, granule deposits also appear between the basal epithelium cell layer and the Bowman layer with various shapes including drop-, crumb-, and ring-shaped [13]. Histopathologically, GCD1 is featured by eosinophilic and rod-shaped hyaline deposits in the cornea, which can be stained bright red by Masson's trichrome [13]. In this study, clinical examinations revealed abundant crumb-shaped and round grayish-white opacities in the subepithelial and anterior stroma layers of the corneas in patients, consistent with features in previously reported *TGFBI* ^p.Arg555Trp^-GCD1 [25]. In previous reports, mutations in the *TGFBI* gene including p.Val113Ile, p.Asp123His, p.Arg124Ser, p.Ser516Arg, p.Arg555Trp, and p.Leu559Val had been described as being involved in GCD1 development [24, 26–29]. In this study, a presumably recurrent heterozygous missense mutation (c.1663C > T, p.Arg555Trp) in the *TGFBI* gene, predicted deleterious by bioinformatics tools, was identified in a three-generation Hui-Chinese family with autosomal dominant inherited GCD1. Intriguingly, the parents of the patient I : 2 were deceased over 75 years of age and were described as unaffected visual acuity by the family members, speculating that the p.Arg555Trp mutation in this family was a *de novo* mutation. To our knowledge, this is the first report of the *TGFBI* c.1663C > T (p.Arg555Trp) mutation in Hui-Chinese, though there is a high number of reported GCD1 cases from China [7, 16, 17]. The discovery of the *TGFBI* gene c.1663C > T (p.Arg555Trp) mutation in several distinct ethnic groups and some families existing in a certain population suggests that both hotspot mutation and founder effect should be considered for the mutation.

The *TGFBI* gene, located at chromosome 5q31.1, contains 17 exons and encodes a 683-amino acid extracellular matrix protein (TGFBIp) with a molecular weight of 68 kDa [25, 30]. TGFBIp contains an N-terminal cysteine-rich EMILIN-like domain, four consecutive and highly homologous fascilin 1 (FAS1) domains, and a C-terminal arginine-glycine-aspartate acid motif [18, 31]. In human corneas, TGFBIp primarily expresses in the epithelium, Bowman's membrane, stroma, and endothelial cell layers, which suggests that it plays crucial roles in corneal damage repair and extracellular matrix maintenance [13, 31]. High levels of TGFBIp expression were associated with postnatal cornea maturation during the early stage of life [31]. The progressive accumulation of insoluble deposits of mutant proteins in the cornea is involved in *TGFBI*-associated CDs, such as GCD1 [31]. Although the precise pathogenesis of *TGFBI* gene mutations resulting in CDs remains to be illustrated, abnormal folding and location alteration of mutant proteins have been proposed as the central mechanism for *TGFBI*-related CDs [13]. It appears that mutations are also likely to change protein degradation pathways and impact structure and stability of aggregated proteins [25]. However, pathogenic mutations in the *TGFBI* gene were reported to have no effect on protein secretion, indicating that mutant proteins escaped from protein secretion regulation system monitoring [32]. Although most of *TGFBI* gene mutations related to CDs are heterozygous, several patients with homozygous mutations have more severe phenotypes, indicating potential toxic functions with a dose-response effect [28].

The Arg555 is located in the fourth FAS1 core domain of the TGFBIp, which is very susceptible to proteolysis [33]. The TGFBIp with the p.Arg555Trp mutation accumulated as crystalloid deposits in GCD1 patient cornea, indicating that the mutation disrupted normal proteolytic degradation by reducing electrostatic repulsion levels [33]. Compared to the wild-type protein, the mutant protein is more stable under physiological pH [34]. The p.Arg555Trp mutation of TGFBIp was also reported to promote human corneal epithelial cells apoptosis by activating the α3*β*1 integrin-related pathway, indicating that it was likely to affect TGFBIp-α3*β*1 integrin interactions [35].

There are no effective approaches to prevent or cure *TGFBI*-related CDs. Although corneal transplantation has been the recommended treatment, a major limitation is the recurrence of posttransplantation corneal deposits [36]. Thus, developing a therapeutic strategy that focuses on preventing mutant TGFBIp deposition by reducing its expression and/or increasing its degradation is likely to be achieved in treatment of *TGFBI*-related CDs. Recently, gene therapy has developed new insights into the treatment of several genetic diseases [36]. *In vitro*, decreasing mutant TGFBIp expression with an allele-specific nature siRNA and correcting mutant DNA in *TGFBI*-mutant cells with site-specific genome editing technologies seem to provide promising approaches for *TGFBI*-linked CDs [37, 38].

Taken together, this study demonstrates that a heterozygous c.1663C > T (p.Arg555Trp) mutation in the *TGFBI* gene is responsible for GCD1 in a Hui-Chinese family, which will be of great help in genetic counseling for this family. Generation of animal models expressing the p.Arg555Trp mutant TGFBIp is likely to reveal the pathogenic mechanisms of GCD1 and shed new light on the development of experimental therapy for this disorder.

## Figures and Tables

**Figure 2:**
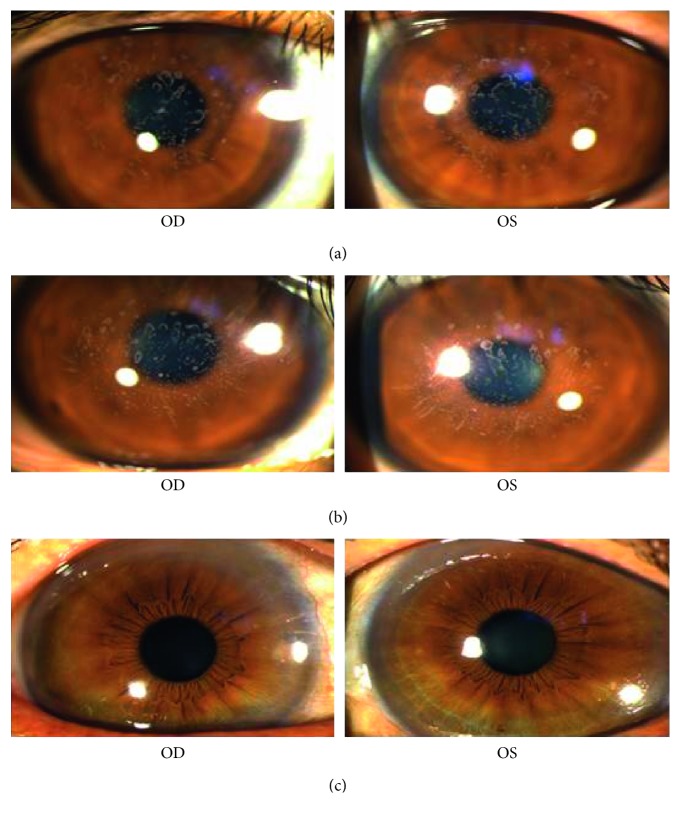
Slit-lamp examinations of the Hui-Chinese family members. The patients II : 1 (a) and III : 1 (b) showed bilateral abundant multiple crumb-shaped and round grayish-white opacities in their central corneas, indicating a GCD phenotype in the family. (c) The unaffected family member (I : 1) showed bilateral normal corneas. OD, right eye; OS, left eye.

## Data Availability

The data used to support the findings of this study are included within the article.
